# The Good Physician

**Published:** 1864-04

**Authors:** 


The Good Physician.—It is beyond our duty to notice the abounding charity of the community unless it take some distinctly medical form. It is only the more agreeable to us, herefore, when we find such distinctly medical shape given to charity as to call for an allusion to it by us. The great amount of efficient charity associated somehow or other with medicine is flattering to the profession. Surely there never was a time when so much will on the part of the rich to help the poor existed as now. If only sound method and true principle could be made available for the guidance of this feeling, it would be impossible to over-estimate the degree to which the character of charity would be elevated, and its utility increased. There is much of mere laziness in the charity which exhausts itself in pitching a penny to a beggar. The true charity would aim at reaching the deeper evils of which rags are merely the dress; and this is an investigation which implies time and trouble and some skill. The late Archbishop Whately is reported to have thanked God that he never gave a beggar a penny. He was a decided philanthropist, but there was method in his philanthropy. He was a philosopher as well. He looked upon poverty as a thing to be treated on principle, not on impulse. The more summary and direct relief of poverty looks kinder, but is really not so difficult, as it certainly is not so efficient as the more sustained and methodical charity, an instance of which we notice to-day. We can well understand that it has been more extensively published than its author either designed or wished, but we hope that he will bear the pain of finding his well-doing famous for the chance of having his example followed.

M. Victor Hugo, of Hauteville, is in the habit of giving forty "most necessitous" children a dinner once a fortnight. Twenty are dined weekly. The dinner is a sound meal of fresh meat and a small glass of wine. In this procedure M. Hugo is guided by the report of a commission of medical and other scientific men appointed in 1848 by the French Government to consider the causes of such diseases as scrofula, rickets, and impoverishment of blood among the children of the poor. This commission gave it as their opinion that these diseases were caused by the children being almost entire strangers to animal food, and would be entirely prevented by their having a meal of fresh meat once a month. M. Hugo goes beyond the requirements of the report, and gives the needed meal once a fortnight. He has been rewarded by undoubted success—the cure of many and the sensible improvement of nearly all of the fortunate children.

What a respectable act of charity, displaying brains as well as heart! What goodly results! Scrofula and rickets kept at bay; the beauty of childhood, that had else been lost, saved; little limbs that had else become soft and lame, kept straight and strong—helps to the acquisition of the appearance and the sensations of health; the dullness of a monotonous poverty lighted up by the prospect and the pleasure of a fortnightly feast: the strengthening of belief in kindness and friendliness; the respect for wealth so used;—in a word, a healthy physical foundation for any amount of good moral influence. This is the true charity alike of religion and reason. "Let it grow."

